# Etanercept as a new therapeutic option for cytokine release syndrome following chimeric antigen receptor T cell therapy

**DOI:** 10.1186/s40164-021-00209-2

**Published:** 2021-02-19

**Authors:** Lina Zhang, Shuai Wang, Ji Xu, Run Zhang, Han Zhu, Yujie Wu, Liying Zhu, Jianyong Li, Lijuan Chen

**Affiliations:** Department of Hematology, the First Affiliated Hospital of Nanjing Medical University, Jiangsu Province Hospital, Collaborative Innovation Center for Cancer Personalized Medicine, Nanjing, 210029 China

**Keywords:** Tumor necrosis factor -α, Etanercept, Cytokine release syndrome, Chimeric antigen receptor T cell therapy, Multiple myeloma

## Abstract

Cytokine release syndrome (CRS) is the most common toxicity induced by chimeric antigen receptor (CAR) T cell therapy. At present, anti-IL-6 agents including tocilizumab and siltuximab have been applied in the treatment of CRS. However, tocilizumab and siltuximab are expensive and some patients fail to respond to anti-IL-6 therapy, which urges the need for new drugs. In clinical practice, we found some patients with multiple myeloma developed markedly increased levels of tumor necrosis factor (TNF)- α during the CRS period after anti-BCMA CAR T cell infusion. Here we present the successful use of TNF-α inhibitor (etanercept) to cure CRS in three patients. The introduction of etanercept did not alter patients' response to CAR T cell therapy and no adverse event was observed directly related to the administration of etanercept. Furthermore, in vitro experiments confirmed that etanercept did not affect the proliferation and effector function of CAR T cells. Our results indicate that etanercept could be considered as a treatment option for CRS in patients with significantly elevated TNF-α levels.

To the Editor,

Over the past decade, chimeric antigen receptor (CAR) T cell therapy has brought hope to patients with relapsed/refractory multiple myeloma (R/R MM), but toxicities such as cytokine release syndrome (CRS) have emerged as new challenges [1]. The CARTOX Working Group and the NCCN guidelines both recommend anti-IL-6 agents (tocilizumab and siltuximab) and corticosteroids as treatments for CRS [2, 3]. However, improved treatment for CRS remains an unmet clinical need, because the existing drugs are not always sufficiently effective. IL-1R antagonist (anakinra) [4], GM-CSF inhibition (lenzilumab) [5], and plasma exchange [6] have been reported as managements for CRS. Moreover, Lee, et al. [7]. successfully used TNF-α inhibitor (etanercept) to treat grade 3 CRS in a 19-year-old female patient with relapsed Hodgkin lymphoma. This was, however, not sufficient to confirm the therapeutic effects of etanercept because she was treated with etanercept and methylprednisolone simultaneously.

Eight patients with R/R MM were enrolled in our study and received LCAR-B38M (anti-BCMA CAR T cells) infusion from March 2017 to March 2020. The clinical characteristics of patients and details of CAR T cell therapy are summarized in Table 1. Fifty-four cytokines were monitored before and after LCAR-B38M infusion by Luminex in all the patients and the detailed results are reported in Additional file 1: Table S1. We found a significant increase in IL-6, TNF-α, IL-10 and TGF-α during the clinical CRS period. More interestingly, Patient 1, Patient 4 and Patient 8 exhibited markedly elevated levels of TNF-α, which was the reason for using etanercept to treat CRS in these patients.

**Table 1:** Clinical data of patients and details of LCAR-B38M therapy

ID	Gender	Age (years)	Subtype	FISH	Conditioning regimen	CAR^+^T (10^6^ /kg)	CRS onset^a^	CRS grading	TNF-α pre^b^ (pg/mL)	TNF-α post^c^ (pg/mL)	Best response	PFS (months)
Patient 1	M	67	IgA κ	Negative	CTX	0.21	Day 7	1	17.64	204.22	sCR	6
Patient 2	M	63	IgA κ	NA	CTX	0.35	Day 9	1	12.36	130.98	NR	6
Patient 3	F	53	IgG κ	Gain(1q), del(13q), t(4; 14)	CTX	0.46	Day 9	1	12.58	13.69	sCR	18
Patient 4	M	56	IgA κ	Gain(1q), del(13q)	CTX	1.52	Day 8	3	4.27	1728.58	sCR	24
Patient 5	F	56	IgA κ	Negative	CTX	0.41	Day 9	1	< 2.5	< 2.5	sCR	33 +
Patient 6	F	64	IgG-κ	t (14; 16)	FC	0.73	Day 4	2	< 2.5	4.45	sCR	13 +
Patient 7	F	67	κ	Negative	FC	0.65	Day 7	1	< 2.5	< 2.5	CR	9 +
Patient 8	M	63	κ	Gain(1q), del(13q), t(11;14)	FC	0.50	Day 6	1	< 2.5	76.09	sCR	7 +

*M* male, *F* female, *FISH* fluorescence in situ hybridization, *CTX* cyclophosphamide 300 mg/m^2^ daily for 3 day, *FC* cyclophosphamide 250 mg/m^2^ daily and fludarabine 25 mg/m^2^ daily for 3 days, *NA* not available, *TNF* tumor necrosis factor, *PFS* progression-free survival

^a^The starting day of LCAR-B38M CAR T-cell infusion was day 0

^b^TNF-α pre refers to the levels of serum TNF-α before CAR T cell infusion

^c^TNF-α post refers to the peak levels of serum TNF-α after CAR T cell infusion

Patient 1 presented with a fever of up to 39.0℃ on day 7 and was treated with paracetamol. However, his body temperature reached 39.4℃ on the second day and he received a subcutaneous injection of etanercept (25 mg) on day 8 (Fig. 1b). Subsequently, his temperature gradually returned to normal. Patient 4 presented with fever on day 8 and experienced neutropenia (grade 3), increased serum aspartate aminotransferase (grade 3), hypotension (grade 3) and arthralgia (grade 3). On days 10 and 11, he received intravenous tocilizumab (240 mg on day 10 and 240 mg twice a day on day 11) and additional supportive care. However, his symptoms did not improve despite the repeated use of tocilizumab; therefore, we used etanercept (50 mg) on day 11 (Fig. 1c). He responded promptly to this treatment and recovery occurred gradually. Patient 8 developed grade 1 CRS on day 6, which was managed with antipyretics. However, he again developed a fever of up to 38.5℃ on day 14. No evidence of severe infection was present. We considered that the re-emergence of fever was also associated with CRS because the serum IL-6 level also increased. Notably, his serum TNF-α level was more than 30 times higher than the baseline. Therefore, etanercept (25 mg) was given on day 17 and symptoms did not recur again (Fig. 1d).

**Fig. 1:**
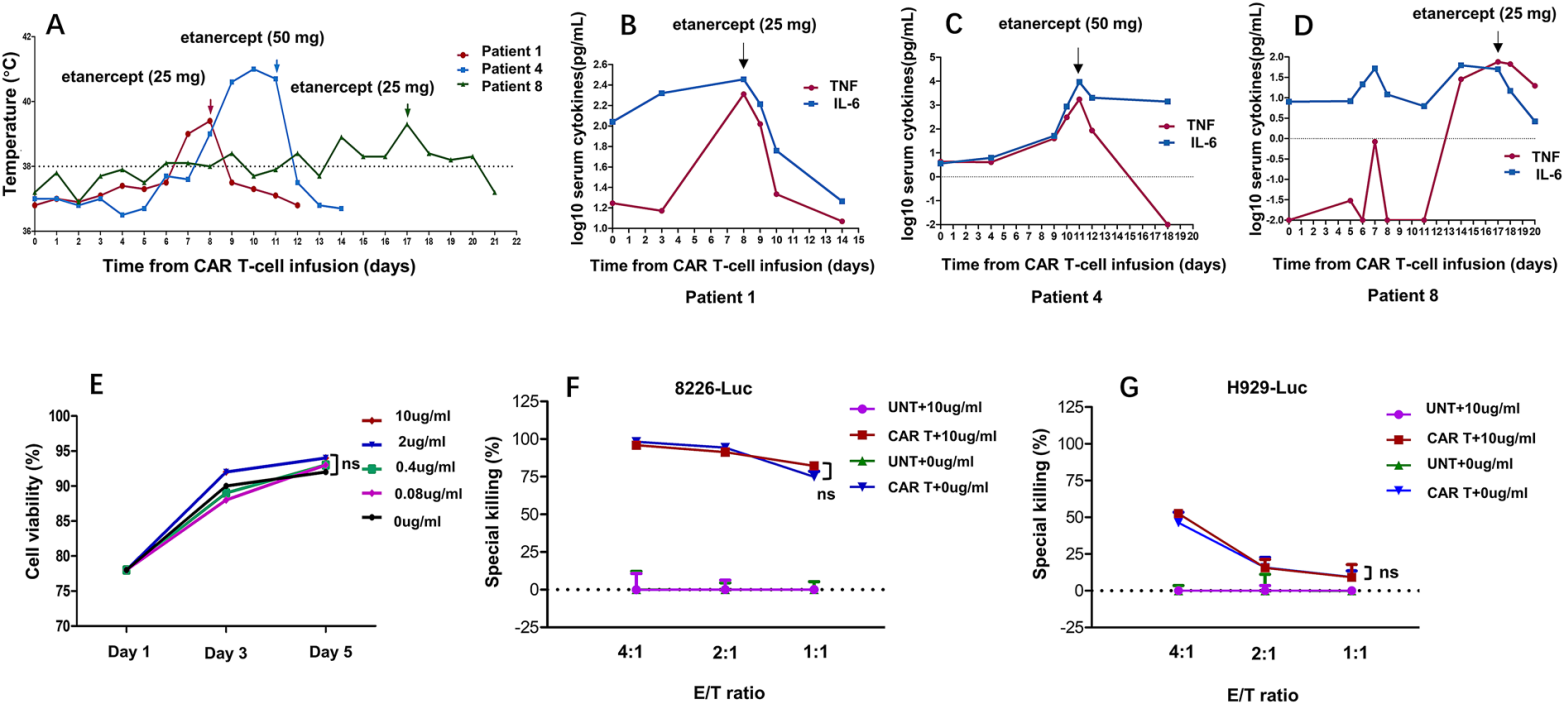
Clinical efficacy of etanercept for cytokine release syndrome and in vitro effects of etanercept on CAR T cells. **a** Maximum temperature for each day of the 3 patients after LCAR-B38M infusion. Arrows indicate the day of etanercept administration. **b–d** IL-6 and TNF-α levels before and after LCAR-B38M infusion in each patient. Cytokine levels are expressed as log10 pg/mL. The values less than the detection limit of serum cytokines were defined as “0” in the statistical analyses. Arrows indicate the day of etanercept administration. **e** Etanercept does not affect CAR T cell proliferation. CAR T cells was treated with 10 μg/mL, 2 μg/mL, 0.4 μg/mL, 0.08 μg/mL, 0 μg/mL etanercept for 5 days. On day 1, day 3 and day 5, cells are collected for cell counting. ns, *P* > 0.05. **f**, **g** Etanercept does not affect CAR T cell effector function. CAR T cells or untransduced T cells (UNT) as effector cells are cocultured with RPMI8226-Luc cells or H929-Luc cells at the effector to target cell ratio of 4:1, 2:1 and 1:1 with or without 10 μg/mL etanercept. ns, *P* > 0.05

To our knowledge, this study is the first attempt to use etanercept monotherapy to treat CRS following CAR T cell therapy. All three patients achieved sCR and we did not observe adverse events directly related to administration of etanercept. Our findings were further supported by in vitro experiments demonstrating that etanercept did not affect CAR T cell proliferation or killing effects on MM cells (Fig. 1e–g). Specific details were provided in Additional file 1.

TNF-α, a major proinflammatory cytokine, is secreted by activated macrophages, monocytes and lymphocytes [8]. Significantly elevated levels of TNF-α have been reported in patients with acute lymphoblastic leukemia who received anti-CD19 CAR T cell infusion [9], which was consistent with our results. Furthermore, the peak level of TNF-α in the serum of patients was considered associated with the severity of CRS [9]. These findings suggest that TNF-α might play an important role in CRS. Etanercept has been widely used in the treatment of rheumatoid arthritis and its efficacy and safety are well established [10]. We suggest that etanercept can be used to manage CRS associated with CAR T cell therapy, and especially recommend for patients suffering from a rapid elevation of TNF-α. Of course, further studies are needed to confirm this suggestion.

## Supplementary Information

**Additional file 1. **Additional tables.

## Data Availability

Data sharing is not applicable to our study.

## Abbreviations

CAR: Chimeric antigen receptor
MM: Multiple myeloma
R/R: Relapsed/refractory
CRS: Cytokine release syndrome
TNF: Tumor necrosis factor
sCR: Stringent complete response
BCMA: B cell maturation antigen.
