# Supplementary material for: Etanercept as a new therapeutic option for cytokine release syndrome following chimeric antigen receptor T cell therapy
Source: Exp Hematol Oncol. 2021 Feb 19;10:16. doi: 10.1186/s40164-021-00209-2 (PMC7893957; doi:10.1186/s40164-021-00209-2)
Supplement: Supplementary file 1 — Additional file 1. Additional tables. [file 40164_2021_209_MOESM1_ESM.docx]

**Materials and Methods**

**Patients**

Patients 1-5 were enrolled in the phase 1 clinical trial of LCAR-B38M (anti-BCMA) CAR T cell therapy for R/R MM from March 2017 to January 2018, which was registered with clinicaltrials.gov (NCT03090659). Patients 6-8 was enrolled in the phase 2 clinical trial of LCAR-B38M from October 2019 to March 2020, which was registered with clinicaltrials.gov (NCT03758417). These trials were approved by the institutional review boards of the First Affiliated Hospital of Nanjing Medical University Ethics Committee. Informed consent was obtained from all patients for the treatment protocol.

**Administration of LCAR-B38M and detection of cytokine**

A cyclophosphamide-based lymphodepleting chemotherapy was used as a conditioning regimen. Cyclophosphamide 250 mg/m^2^ daily and fludarabine 25 mg/m^2^ daily for 3 days or cyclophosphamide 300 mg/m^2^ daily for 3 days was administered. Five days later, patients received LCAR-B38M cell infusion. The starting day of LCAR-B38M CAR T-cell infusion was day 0. Fifty-four cytokines were monitored before and after LCAR-B38M infusion by Luminex in all cases.

**Evaluation of efficacy and toxicities**

Efficacy of LCAR-B38M was evaluated by criteria of response according to the IMWG consensus recommendations. Adverse events were graded following National Cancer Institute Common Terminology Criteria for Adverse Events (NCI-CTCAE v. 4.03). The CRS grade was determined by the criteria reported by the CARTOX working group.

**Lentivirus package and titer**

LentiX-293T cells (Clontech, Takara Bio) were used for lentivirus production. 600-700M cells were seeded into 10-layers CellSTACKs according to the optimized density, and were cultured in a 37°C, 5% CO_2_ incubator overnight. Then cells were transfected with pMDLg.pRRE, pRSV-Rev, pMD.2G and transfer plasmid by using the PEIpro-HQ transfection method. PEI reagent was added into the plasmids mixture at the volume ratio of 3:1. 48-72 hours post-transfection, supernatant was collected and concentrated by chromatography to obtain purified lentivirus. 0.5×10^6^ CHO cells in 2 mL were added to 6 well plates and serially diluted lentivirus were added into each well respectively to initiate the transduction. 3 days later, the cells of each well were collected and stained with BCMA-FITC (ACRO, Cat No. BCA-HF254) for 15min followed by flow cytometry assay to evaluate the virus infection titer.

**CAR T cell preparation**

T cells were purified from patients by Pan T Cell Isolation Kit (Miltenyi Biotec, Cat No.130-096-535). The obtained T cells were stimulated for 48 hours by using T Cell TransAct reagents (Miltenyi Biotec, Cat No.130-111-160) following instructions from the manual. On the day of transduction, 5×10^6^ T cells were transferred into each well of G-Rex 6M plate. Lentivirus was mixed with cell suspension at MOI (multiplicity of infection) of 5. The transduced cells, as well as un-transduced cells (UNT) were allowed for ex vivo culture with TexMACS GMP medium. The prepared cells were then harvested on day 7 post-transduction and cryopreserved in liquid nitrogen.

**Proliferation of CAR T cells**

CAR T cells were thawed and recovered overnight. On the next day, CAR T cells were treated with 10μg/mL, 2μg/mL, 0.4μg/mL, 0.08μg/mL, 0μg/mL etanercept for 5 days. On day 3, the medium were refreshed and added with different concentration etanercept. On day 1, day 3 and day 5, cells were collected for cell counting (Nexcelom Bioscience, Cat No. K2).

**In vitro cytotoxicity co-culture assay**

CAR T cells or untransduced T cells (UNT) as effector cells were cocultured with RPMI8226-Luc cells or H929-Luc cells at the effector to target cell ratio of 4:1, 2:1 and 1:1 with or without 10μg/mL etanercept. After 24h, the cells were added with 100 μL ONE-Glo Firefly luciferase assay reagent mix (Promega, E6120) and incubated at room temperature for 1 min. The remaining live target cells indicated by active luciferase activities were read as Relative Light Unit (RLU) in microplate reader (Tecan Spark 10M). The cytotoxicity of CAR T on target cells were calculated with formula as $Cytotoxicity\%=1-\frac{{RLU}_{sample}-{RLU}_{min}}{{RLU}_{UnT}-{RLU}_{min}} 100\%$.

**Statistics and data**

Results were analyzed using software GraphPad Prism version 5.0. *P* value more than 0.05 was considered significant. Paired comparisons between cytokines before and after CAR T cell infusion were conducted with Wilcoxon paired t-tests. Proliferation and cytotoxicity were compared using ANOVA.

| **Cytokines** | ***P* value** | **Cytokines** | ***P* value** | **Cytokines** | ***P* value** | **Cytokines** | ***P* value** |
| --- | --- | --- | --- | --- | --- | --- | --- |
| **IL-6** | **0.0078** | Flt-3 Ligand | 0.3018 | IL-3 | 0.1305 | Angiopoietin-2 | 0.3392 |
| **TNF-α** | **0.0313** | G-CSF | 0.3758 | IL-33 | 0.2500 | VEGF R1 | 0.4401 |
| GM-CSF | 0.1114 | Eotaxin | 0.7500 | IL-4 | 0.6250 | VEGF R2 | 0.2607 |
| IFN-γ | 0.2493 | IL-1α | Undetectable | IL-5 | Undetectable | VEGF R3 | 0.1460 |
| IP-10 | 0.2933 | IL-1β | 0.4220 | IL-7 | 0.2500 | RAGE | 0.2672 |
| MCP-1 | 0.2149 | IL-6Rα | 0.3151 | PDGF-AA | 0.2682 | CD25 | 0.1210 |
| MIP3-α | 0.3693 | IL-8 | 0.4191 | PDGF-AB | 0.9500 |  |  |
| MIP1-α | 0.3339 | **IL-10** | **0.0171** | RANTES | 0.5730 |  |  |
| MIP1-β | 0.3488 | IL-12 | 0.2142 | **TGF-α** | **0.0213** |  |  |
| CXCL2 | 0.1317 | IL-13 | Undetectable | gp130 | 0.1399 |  |  |
| CX3CL1 | 0.3140 | IL-15 | 0.2214 | TRAIL | 0.1371 |  |  |
| CXCL1 | 0.2561 | IL-17A | 0.3025 | VEGF | 0.0679 |  |  |
| CCL19 | 0.2500 | IL-17E | 0.3467 | FGF-basic | 0.2500 |  |  |
| IFN-α | 0.0819 | IL-18 | 0.2634 | MIG | 0.1764 |  |  |
| IFN-β | Undetectable | IL-1RA | 0.2683 | CCL22 | 0.1596 |  |  |
| EGF | 0.1290 | IL-2 | 0.0672 | Angiopoietin-1 | 0.1937 |  |  |

Table S1 Serum cytokine profiles in eight patients.

Table S2 Serum cytokine profiles of the three patients who received anti-TNF-α therapy.

| **ID** | **Time (day)** | **TNF-α (pg/mL)** | **IL-6 (pg/mL)** | **IL-2 (pg/mL)** | **IL-4 (pg/mL)** | **CRP (mg/L)** | **SF (ng/ml)** |
| --- | --- | --- | --- | --- | --- | --- | --- |
| Patient 1 | B | 17.64 | 109.76 | 24.96 | 24.73 | 71.80 | 730.40 |
|  | 3 | 14.87 | 208.63 | 22.58 | 19.60 | 53.90 | 757.60 |
|  | 8 | 204.22 | 285.26 | 24.61 | 23.37 | 48.70 | 1085.60 |
|  | 9 | 104.58 | 163.22 | 20.97 | 20.20 | NA | NA |
|  | 10 | 21.60 | 57.50 | 23.92 | 24.04 | 76.40 | 1817.00 |
|  | 14 | 11.72 | 18.39 | 19.43 | 17.60 | NA | 2290.00 |
| Patient 4 | B | 4.27 | 3.60 | <2.50 | <2.50 | <3.02 | 98.20 |
|  | 4 | 4.08 | 6.21 | <2.50 | <2.50 | <3.02 | 83.70 |
|  | 9 | 40.24 | 51.78 | 4.96 | <2.50 | NA | NA |
|  | 10 | 308.79 | 866.28 | <2.50 | <2.50 | 55.30 | 1366.90 |
|  | 11 | 1728.58 | 9278.33 | <2.50 | <2.50 | 46.00 | NA |
|  | 12 | 86.39 | 2039.07 | <2.50 | <2.50 | NA | 5173.00 |
|  | 18 | <2.50 | 1397.47 | <2.50 | <2.50 | NA | NA |
| Patient 8 | B | <2.50 | 7.99 | <2.50 | 0.48 | 3.27 | 282.60 |
|  | 5 | 0.03 | 8.27 | 4.42 | 1.27 | 10.20 | 373.00 |
|  | 6 | <2.50 | 21.33 | 8.10 | <2.50 | NA | NA |
|  | 7 | 0.84 | 52.07 | 1.89 | 1.21 | 15.10 | 557.30 |
|  | 8 | <2.50 | 12.08 | 0.60 | 1.34 | 30.60 | 486.50 |
|  | 11 | <2.50 | 6.20 | <2.50 | 0.38 | 36.00 | 458.00 |
|  | 14 | 28.42 | 62.23 | 0.60 | <2.50 | 7.39 | 379.00 |
|  | 17 | 76.09 | 49.90 | 0.60 | 0.71 | 21.70 | 1131.60 |
|  | 18 | 66.98 | 14.75 | 0.19 | 0.59 | 18.80 | 1396.50 |
|  | 20 | 19.58 | 2.64 | 1.71 | 0.77 | NA | NA |

**Abbreviation:** CRP: C-reactive protein; SF: serum ferritin; NA: not available; B: baseline.
